# Immediate Care of Open Extremity Fractures: Where Can We Improve?

**DOI:** 10.1155/2014/807694

**Published:** 2014-01-21

**Authors:** R. Walton, J. Manara, S. E. Elamin, I. Braithwaite, E. Wood

**Affiliations:** Mersey Deanery, Regatta Place, Summers Road, Brunswick Business Park, Liverpool L3 4BL, UK

## Abstract

Clear guidelines are set by the British Orthopaedic Association (BOA) and British Association of Plastic, Reconstructive and Aesthetic Surgeons (BAPRAS) on the preoperative management of open fractures. This as well as the clinical consequences of poor management of open fractures means the patient workup for surgery is important as well as the timing of surgery. Experience suggests few patients are managed 100% as per the guidelines and we look to test this hypothesis. A retrospective analysis was undertaken of all open long bone fractures (total 133), excluding hand injuries, which presented to a district general hospital over a 5-year period. The implementation of 7 defined key tasks for initial management was recorded. 101 cases were eligible, with the majority of cases (71.4%) having initial orthopaedic assessment outside normal working hours. The mean number of tasks completed was 3.23/7. Assessment out of hours was associated with less tasks being implemented but doctor seniority and the presence of polytrauma made no difference to the quality of acute care. Staff involved in the acute care of open fractures require targeted education to improve the delivery of initial preoperative care. We recommend that other centres assess their performance against this data.

## 1. Introduction

There have recently been changes to both the British Orthopaedic Association (BOA) and British Association of Plastic, Reconstructive and Aesthetic Surgeons (BAPRAS) on the preoperative management of open fractures [1, 2] These changes have suggested that the initial surgical debridement and stabilisation of open fractures do not have to occur within 6 hours of injury, as previously advised [3]. Initial, preoperative steps in management have been clearly outlined within these guidelines, and it is particularly important that all cases receive a complete package of care on admission in light of the increased number of cases undergoing delayed surgical intervention.

Both the 1997 and 2009 BOA/BAPRAS guidelines have recommended that patients should be assessed for compartment syndrome and vascular injury [3, 4]. Both sets of guidelines also state that tetanus immunisation status should be recorded, IV antibiotics administered, and that the wound should be photographed [3, 4]. Both guidelines state that the wound should be sealed from the environment. While the 1997 guidelines advise a wound swab should be sent, the 2009 guidelines do not discuss this [3, 4]. There is a large amount of evidence to support the use of IV antibiotics in particular [4–9].

Previous literature on standards of acute open fracture care has investigated compliance with the now historic “6-hour rule” and the time taken to achieve soft tissue cover [3, 9]. No literature was found to illustrate trends in implementation of the basic initial preoperative management steps.

The aim of this study was to investigate the current adequacy of immediate acute open fracture care. Our hypothesis from clinical experience is that these guidelines are not being fully followed. By looking at the following variables we look to test our hypothesis and thereby also assess the adequacy of immediate open fracture care: the influence of injury severity, doctor seniority, polytrauma, and time of admission. By describing current management trends, it will be possible to highlight areas where improvement is necessary and to suggest changes to bring this about. This data should also encourage other centres to audit acute open fracture care.

## 2. Patients and Methods

We obtained details of all patients admitted to a typical district general hospital with a diagnosis of open fracture between December 2002 and July 2007. The cases were found by the use of ICD-10 codes. Open fractures of all long bones, excluding hand fractures, were included.

Data was collected retrospectively from case notes and the hospital computer database (MEDITECH Workstation 4). Patient demographics were recorded, alongside details of the injury and coexisting injuries. Time of injury, arrival at accident and emergency, and time of initial orthopaedic review were recorded; times between 08:00 and 17:00 were defined as “within working hours.” We did not look at the delay from injury to arrival in the emergency department or from referral to orthopaedic review as we were concentrating on the management aspects rather than timing.

Seven key tasks to be performed in the initial orthopaedic review were defined as per British Orthopaedic Association guidelines. All cases should have clear documented evidence that an assessment for compartment syndrome was carried out. All cases should have clear documented evidence that an assessment for vascular injury was undertaken. The patient's tetanus immunisation status should be recorded in all cases. A photograph of the wound, or clear documentation that a photograph was taken, should be present in all case notes. A wound swab should be sent to microbiology from all wounds (either clear documentation of a swab being sent or evidence on the hospital computer database of a swab being processed). All patients should receive IV antibiotics (either documented evidence in the case notes or a prescription at the time of admission on old drug charts present in the case notes). Clear documented evidence that an antiseptic dressing was applied to all wounds. On top of this the grade of orthopaedic doctor assessing the patient was recorded to determine whether this had an effect on management. This was either a senior house officer (SHO) or specialist registrar (SPR). SHOs are more junior and are not always focused on a career in orthopaedics but are on site 24 hours per day and are usually the doctor the emergency department refers patients to. Specialist registrars are a more senior grade of doctor and are specialists in orthopaedics; however they may not be resident outside working hours. The data collected was entered into a standardised pro forma. Each key task was analysed separately and the number of tasks performed was also analysed as a whole. The proportion of cases where each individual task was undertaken was analysed with the Pearson Chi Square test. In situations when the number of cases where a task was undertaken equalled less than 5, the Fisher exact test was used. When analysing the total number of tasks performed in a group of patients, Student's *t*-test was used. The level of statistical significance was taken as *P* = 0.05.

## 3. Results

133 patients were initially included. 32 patients were admitted acutely to another centre and subsequently transferred. These patients were therefore excluded, leaving a study population of 101 patients, who sustained 105 open fractures.

**Figure 1:**
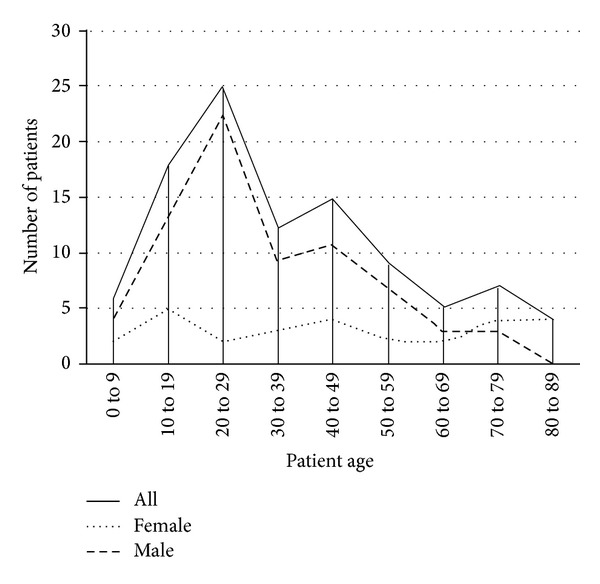
Age of patients in study group.

28 patients were female (27.7%), compared to 73 males (72.3%). Mean age was 35.9 years (range 4–86). When analysed by gender, age distribution followed different patterns. Female patients had a mean age of 45.6 years (range 4–86) and male patients 32.2 years (range 4–74). The higher mean age of female patients was statistically significant (*P* = 0.011, Student's *t*-test).  Figure 1 shows a graphical representation of trends in patient age.

**Figure 2:**
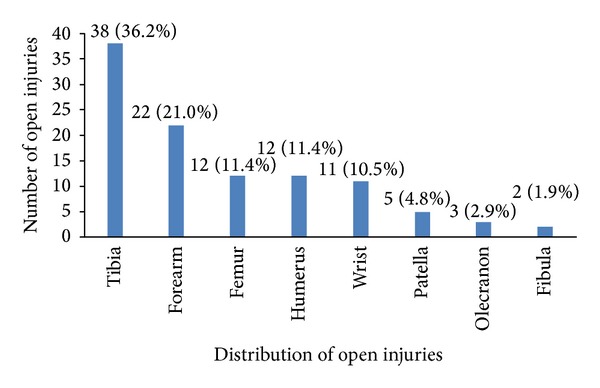
Skeletal distribution of open fractures.

The 105 open fractures sustained by the study population most frequently involved the tibia (38/105 = 36.2%) or forearm (22/105 = 21.0%).  Figure 2 shows the skeletal distribution of all open injuries.

**Figure 3:**
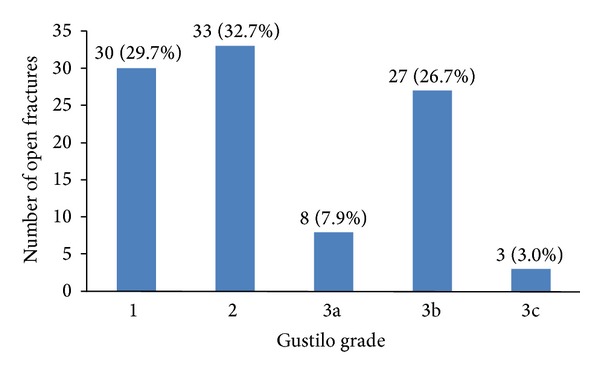
Distribution of injury severity.

All case notes had either a direct record of Gustilo classification or adequate information recorded to classify the injury retrospectively. Four patients had 2 open injuries; in this case, the more significant of the 2 injuries has been classified and included in subsequent analyses.  Figure 3 shows the distribution of injury severity, as described by Gustilo classification.

In 74 cases (73.3%) the open fracture was the only injury sustained, in comparison to 27 cases (26.7%) where polytrauma was sustained.

**Figure 4:**
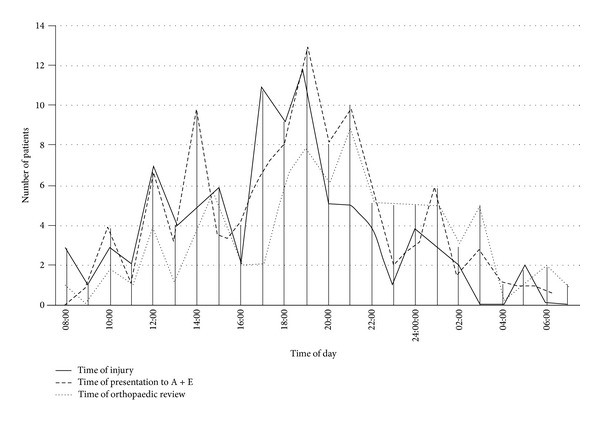
Trends in time of injury, presentation to the emergency department, and initial orthopaedic review.

The majority of the study population sustained their injury, presented to the emergency department, and had an initial orthopaedic review outside normal working hours. 59 patients (58.4%) sustained injury outside this time period. 62 (61.4%) presented outside working hours to the emergency department. Of 84 patients where a time was recorded for initial orthopaedic review, this was outside normal working hours in 60 cases (71.4%).  Figure 4 shows the time distribution of injury, presentation to the emergency department, and initial orthopaedic review.

Injury severity did not differ in patients who had their initial orthopaedic review either within or outside normal working hours. In those reviewed within working hours, Gustilo grade 3 injuries represented 8/23 (34.8%). Gustilo grade 3 injuries represented 23/60 (38.3%) of cases reviewed outside working hours. Grade of doctor performing the initial orthopaedic review was recorded in 98/101 cases (97.0%). In 82/98 cases (83.7%), this was a senior house officer, while in 16/98 (16.3%) it was a specialist registrar. Of the patients reviewed by a specialist registrar, proportionally more had Gustilo grade 3 injuries (8/16 = 50%), compared to those patients reviewed by senior house officers (29/82 = 35.4%). This difference did not reach statistical significance.

Table 1 provides a summary of results, which are subsequently discussed. 22/101 patients (21.8%) had documented evidence of an assessment for compartment syndrome. One patient in the series developed a compartment syndrome which required fasciotomy. This patient had sustained polytrauma, with a Gustilo grade 3b open tibial fracture. He was not assessed for compartment syndrome during the initial review.

10/63 (15.9%) of Gustilo grade 1 and 2 injuries were assessed for compartment syndrome, in comparison to 12/38 (31.6%) of Gustilo grade 3 injuries. This difference approached significance (*P* = 0.064). Seniority of doctor did not have any effect. There was no statistical difference in the number of polytrauma patients assessed for compartment syndrome, in comparison to those with an isolated injury. The timing of initial orthopaedic review did not affect the proportion of patients who were assessed for compartment syndrome.

95/101 patients (94.1%) had a documented assessment of vascular status. Three patients had a vascular injury, which was recognised on admission in all cases. Injury severity, doctor seniority, the presence of polytrauma, and timing of assessment had no statistical effect on the proportion of patients who underwent assessment for vascular injury.

Tetanus status was recorded in 30/101 cases (29.7%). There was a trend approaching significance (*P* = 0.053) towards a higher rate of recording of tetanus status in Gustilo grade 3 injuries (16/38 = 42.1%). Gustilo grade 1 and 2 injuries had tetanus status recorded in 15/63 cases (23.8%). Doctor seniority, polytrauma, and timing of assessment had no effect.

Photographs were taken of 19/101 wounds (18.8%). 5/63 (7.9%) Gustilo grade 1 and 2 injuries had a photograph, compared to 14/38 (36.8%) Gustilo grade 3 injuries. This difference was statistically significant (*P* = 0.003). Doctor seniority, polytrauma, and timing of assessment had no effect.

Swabs were sent in 5/101 cases (5.0%). In Gustilo type 3 injuries swabs were sent in 4/38 (10.5%), while in Gustilo type 1 and 2 injuries this figure was 1/63 (1.6%). This difference approached significance (*P* = 0.065). There was no difference in the frequency of cases where swabs were sent according to doctor seniority, polytrauma, and timing of assessment.

Antibiotics were given in 84/101 cases (83.2%). 36/38 (94.7%) Gustilo grade 3 injuries received antibiotics. This figure was 48/63 in Gustilo grade 1 and 2 injuries (76.2%). This difference reached significance (*P* = 0.016). Doctor seniority, polytrauma, and timing of assessment had no effect on the rate of antibiotic administration.

Antiseptic dressings were applied in 69/101 injuries (68.3%). Patients assessed within working hours had an antiseptic dressing in 20/23 cases (87.0%), while those patients assessed outside working hours had an antiseptic dressing in 40/60 cases (66.7%). This difference approached significance (*P* = 0.064). Injury severity, doctor seniority, and polytrauma had no effect.

When all 7 tasks were analysed as a whole, the mean number of tasks completed per patient was 3.23. Only 1/101 cases had all 7 tasks completed. The mean number of tasks completed per patient was higher (3.82) in Gustilo grade 3 injuries than in Gustilo grade 1 and 2 injuries (2.83). The difference was statistically significant (*P* = 0.0004, Student's *t*-test). In patients assessed within working hours the mean number of tasks completed was higher (3.74) than in those assessed outside working hours (3.18). The difference was also statistically significant (*P* = 0.004). Seniority of doctor had no effect (*P* = 0.276), likewise polytrauma (*P* = 0.298).

## 4. Discussion

The majority of patients studied with open long bone fractures were males in their second, third, and fourth decades, which is likely to be secondary to the increased rates of trauma in males in these age groups (75% of major trauma patients are male) [10, 11]. Female patients presented over a wider age range and had a significantly higher mean age, most likely due to the higher rate of fragility fractures in these patients [12]. Most cases sustained isolated open fractures, but a significant proportion of injuries were in the context of polytrauma. Patterns of injury were such that the majority of cases had their initial orthopaedic review outside the hours of 08:00 to 17:00. Time of presentation did not affect injury severity. The grade of orthopaedic doctor performing the initial orthopaedic review was most frequently a senior house officer again likely to be secondary to the fact most presented out of hours when senior cover is not as common.

Implementation rates of the 7 key management tasks left a substantial opportunity for improvement. The least frequently completed tasks were sending wound swabs (5.0%), taking a photograph of the wound (18.8%), documenting assessment of compartment syndrome (21.8%), and documenting of tetanus immunisation status (29.7%). These are likely to represent staff being unaware of the necessity of these investigations and treatment. In contrast, the following key tasks were undertaken in a higher proportion of cases: assessment for vascular injury (94.1%), administration of IV antibiotics (83.2%), and application of an antiseptic dressing (68.3%). These treatment options are more intuitive which may represent the improved rates. Although these tasks were undertaken more frequently, there was still scope for improvement.

The quality of acute care was affected by injury severity. Gustilo type 3 injuries were statistically more likely to have a photograph and receive IV antibiotics. The increased performance of key tasks in Gustilo type 3 injuries suggests that juniors were able to recognise the severity of these injuries. It also suggests that underperformance in acute care of Gustilo type 1 and 2 injuries was not due to educational deficiencies alone. This finding is supported by the decreased number of key tasks performed in cases which were assessed out of hours. It should be remembered that all open fractures need to be taken seriously and management steps should not be omitted, regardless of injury severity.

There was a nonsignificant trend for these cases to be more commonly assessed for compartment syndrome, have their tetanus status recorded, and have an antiseptic dressing applied. More key tasks were completed in each Gustilo type 3 injury in comparison to type 1 and 2 injuries. The improvement in acute care provision was not transmitted to cases where polytrauma was sustained. Seniority of doctor did not affect the implementation rates of any of the key tasks or the number of key tasks completed per case. Those patients seen within working hours had significantly more tasks completed. There was no significant difference in any specific task.

These results confirm our hypothesis that the guidelines are not being fully followed. It is conceivable that junior orthopaedic staff do not perceive compartment syndrome as a problem in open fractures. There may be a feeling that the compartment has been decompressed due to the open injury. This belief is wrong and should be discouraged actively. Keating et al. found the rate of compartment syndrome to be 6% in open tibial fractures [8]. Juniors should be educated the importance of looking for and recognising compartment syndrome in this setting. Antibiotic administration in open fractures is of paramount importance. Orthopaedic departments should ensure that all staff who may be involved in the acute care of open fractures are aware of the importance of antibiotic administration.

The reason for underperformance in taking wound swabs, applying antiseptic dressings, and documenting tetanus status may arise from junior orthopaedic staff considering these tasks to be the responsibility of other team members. This is not the case and juniors should be reminded that although a team approach to management is to be commended, they should ensure that these important management steps are achieved in all cases. A digital camera with instructions should be readily available in all units accepting trauma. Juniors should be familiarised with the equipment and consent procedures for photography when starting work in the unit.

Involvement of specialist registrars occurred in relatively few open fractures. This is likely to be related to the high number of injuries that occurred out of hours. Although increasing seniority of doctor performing the initial orthopaedic assessment did not increase the quality of acute care, specialist registrars should be available at least in an advisory capacity. This is of particular importance in light of decreasing experience of junior trainees in the current climate of limited working hours. Improved education of all trainee grades should be recommended. The reason this study was important is that open fractures are common and if not managed appropriately can have negative outcomes for the patient. It is because of these reasons that there are guidelines and it is important all doctors involved in managing orthopaedic patients are aware of these guidelines and the importance of following them.

This study was limited by reliance on documentation in case notes to calculate the implementation of some of the key tasks. While it is possible that task implementation could be underestimated as a result, it should be stressed that documenting assessment for compartment syndrome and vascular status is of extreme importance and should be carried out in all cases. Full documentation is essential from both a clinical and medicolegal point of view. It should be highlighted that in the case of wound swabs, which had the lowest compliance level, case note documentation was not relied on exclusively.

## 5. Conclusion 

Our results show significant opportunity for improvement in the acute care of open fractures. They do show that in the patients who present with grade 3 Gustilo classification fractures are more likely to have more key tasks completed and so better compliance with the guidelines. Given BOA and BAPRAS guidelines are evidence-based, improved outcomes should be assumed to correlate with the number of tasks completed. Given Gustilo 3 fractures are more serious presenting conditions this may reflect the clinicians intuitive response to perform more investigations and spend more time ensuring the patient is appropriately managed, which may explain our results. The results suggest certain tasks such that intravenous antibiotics are given in nearly all cases; however certain tasks such as swabs of the wound or photos of the wound are much less likely to be performed. These tasks are done with increasing frequency in Gustilo 3 fractures. Certain tasks such as assessment of compartment syndrome were only documented in 15% of cases; however it is likely that this will have taken place in the majority of cases without findings being documented. This is important because the consequences of a missed compartment syndrome are serious and documentation is essential from a medicolegal point of view.

The implementation of all key tasks could be improved, by junior and senior orthopaedic doctors. We recommend increased attention is paid to providing education of junior orthopaedic staff in this field. This should include specific tasks that must be undertaken in all open fractures. There is no previous literature to compare our findings against and we recommend that units audit acute open fracture care to improve performance at both local and national levels.

This study is meaningful as open fractures are a common presenting condition to orthopaedic surgeons. There are clear guidelines set by the BOA and BAPRAS which are not being fully followed and by highlighting this we hope this will improve compliance amongst junior doctors involved in the acute care of this group of patients. We also hope this will encourage more senior orthopaedic surgeons to reinforce these principles to juniors both by clinical practice and formal teaching.

## Figures and Tables

**Table 1:** Summary of results.

Key task	Polytrauma versus isolated injury	Assessed by senior house officer versus specialist registrar	Gustilo type 1 + 2 versus type 3 injuries	Whole sample
Assessed for compartment syndrome	4/27 (14.8%) versus 18/74 (24.3%) *P* = 0.306	18/82 (21.2%) versus 4/16 (25.0%) *P* = 0.507	10/63 (15.9%) versus 12/38 (31.6%) *P* = 0.064*	22/101 (21.8%)
Assessed for vascular injury	27/27 (100%) versus 68/74 (91.9%) *P* = 0.146	76/82 (92.7%) versus 16/16 (100%) *P* = 0.333	60/63 (95.2%) versus 35/38 (92.1%) *P* = 0.44	95/101 (94.1%)
Tetanus status recorded	6/27 (22.2%) versus 24/74 (32.4%) *P* = 0.320	26/82 (31.7%) versus 3/16 (18.6%) *P* = 0.235	15/63 (23.8%) versus 16/38 (42.1%) *P* = 0.053*	30/101 (29.7%)
Photograph of wound	5/27 (18.5%) versus 14/74 (18.9%) *P* = 0.606	15/82 (18.3%) versus 4/16 (25.0%) *P* = 0.375	5/63 (7.9%) versus 14/38 (36.8%) *P* = 0.003^#^	19/101 (18.8%)
Wound swab sent	3/27 (11.1%) versus 2/74 (2.7%) *P* = 0.117	4/82 (4.9%) versus 1/16 (6.3%) *P* = 0.598	1/63 (1.6%) versus 4/38 (10.5%) *P* = 0.065*	5/101 (5.0%)
IV antibiotics given	23/27 (85.2%) versus 61/74 (82.4%) *P* = 0.502	68/82 (82.9%) versus 15/16 (93.8%) *P* = 0.247	48/63 (76.2%) versus 36/38 (94.7%) *P* = 0.016^#^	84/101 (83.2%)
Antiseptic dressing applied	16/27 (59.3%) versus 53/74 (71.6%) *P* = 0.237	55/82 (67.1%) versus 12/16 (75.0%) *P* = 0.380	42/63 (66.7%) versus 36/38 (94.7%) *P* = 0.517	69/101 (68.3%)
Number of tasks completed/7	3.11 versus 3.27 *P* = 0.298	3.22 versus 3.60 *P* = 0.276	2.83 versus 3.82 *P* = 0.0004^#^	3.23

^#^Statistically significant result.

*Trend but not statistically significant.
